# “SMALL BITES” QUALITY IMPROVEMENT: A SLEEP INTERVENTION FOR RESIDENTS WITH DEMENTIA IN THE NURSING HOME “NEW NORMAL”

**DOI:** 10.1093/geroni/igad104.1639

**Published:** 2023-12-21

**Authors:** A Lynn Snow, Megan McCullough, Robert Morgan, Rosa Baier, Ellen McCreedy, Kathy Richards, Liam Fry, Christine Hartmann

**Affiliations:** University of Alabama, Tuscaloosa, Alabama, United States; University of Massachusetts Lowell, Lowell, Massachusetts, United States; University Of Texas Health Science Center, Houston, Texas, United States; Brown University School of Public Health, Providence, Rhode Island, United States; Brown University School of Public Health, Providence, Rhode Island, United States; University of Texas at Austin, Austin, Texas, United States; University of Texas at Austin, The, Austin, Texas, United States; VA Bedford Healthcare System, Bedford, Massachusetts, United States

## Abstract

Together with three nursing home (NH) corporations, we co-designed and adapted a “small bites” approach to pragmatic clinical trial (PCT) implementation that was successful even during the pandemic height. Our pilot PCT to improve sleep for nursing home residents with dementia began with the start of the pandemic, with its increased resident acuity and resident care burden, increased workforce shortages and instability, and need for increased leadership involvement. Using co-design principles, we reviewed all procedures with our first NH and identified ways to reduce staff burden by “bite-sizing” the as much implementation protocol as much as possible (e.g., serial 5-minute staff trainings). We implemented revised procedures in all three pilot NHs. To identify adaptations, we used a modified version of the Framework for Reporting Adaptations and Modifications-Enhanced (FRAME) to code meeting minutes, researcher fieldnotes about adaptations, and 35 group interview transcripts with the leadership/champion teams. We also coded for feasibility, acceptability, and sustainability. We used an iterative, rapid content analytic approach to qualitative analyses. Most adaptations occurred in the pre-implementation phase, although some occurred in the pilot implementation phase. Adaptations concerned reducing staff burden, such as moving from in-person visits to a fully virtual approach that used 30-minute once-a-week project meetings and streamlining how staff completed measures. The resulting intervention package worked in all corporations on all levels: feasibility, outcome, and sustainability. Necessity being the mother of invention, we landed on a better process than originally planned, one that may be valuable for the wider nursing home research community.

